# Phase I-metabolism studies of the synthetic cannabinoids PX-1 and PX-2 using three different in vitro models

**DOI:** 10.1007/s11419-021-00606-6

**Published:** 2021-12-30

**Authors:** Patrick Dahm, Andreas Thomas, Markus A. Rothschild, Mario Thevis, Katja Mercer-Chalmers-Bender

**Affiliations:** 1 Institute of Legal Medicine, Faculty of Medicine, University of Cologne, Melatengürtel 60/62, 50823 Cologne, Germany; 2 Institute of Biochemistry, German Sport University Cologne, Am Sportpark Müngersdorf 6, 50933 Cologne, Germany; 3 Institute of Forensic Medicine, Department of Biomedical Engineering, University of Basel, Pestalozzistr. 22, 4056 Basel, Switzerland; 4 Institute of Legal Medicine, University Hospital Münster, Röntgenstr. 23, 48149 Münster, Germany; 5 Department of Physics and Chemistry of Materials, Faculty of Medicine/Dental Medicine, Danube Private University, Krems, Austria

**Keywords:** pHLM, CYP isoenzymes, *Cunninghamella elegans*, In vitro metabolism, PX-1, PX-2

## Abstract

**Purpose:**

Synthetic cannabinoids (SCs), highly metabolized substances, are rarely found unmodified in urine samples. Urine screening relies on SC metabolite detection, requiring metabolism knowledge. Metabolism data can be acquired via in vitro assays, e.g., human hepatocytes, pooled human liver microsomes (pHLM), cytochrome P450 isoforms and a fungal model; or in vivo by screening, e.g., authentic human samples or rat urine. This work describes the comprehensive study of PX-1 and PX-2 in vitro metabolism using three in vitro models. 5F-APP-PICA (PX-1) and 5F-APP-PINACA (PX-2) were studied as they share structural similarity with AM-2201, THJ-2201 and 5F-AB-PINACA, the metabolism of which was described in the literature.

**Methods:**

For SC incubation, pHLM, cytochrome P450 isoenzymes and the fungal model *Cunninghamella elegans* LENDNER (*C. elegans*) were used. PX-1 and PX-2 in vitro metabolites were revealed comprehensively by liquid chromatography–high-resolution mass spectrometry measurements.

**Results:**

In total, 30 metabolites for PX 1 and 15 for PX-2 were detected. The main metabolites for PX-1 and PX-2 were the amide hydrolyzed metabolites, along with an indole monohydroxylated (for PX-1) and a defluorinated pentyl-monohydroxylated metabolite (for PX-2).

**Conclusions:**

CYP isoforms along with fungal incubation results were in good agreement to those obtained with pHLM incubation. CYP2E1 was responsible for many of the metabolic pathways; particularly for PX-1. This study shows that all three in vitro assays are suitable for predicting metabolic pathways of synthetic cannabinoids. To establish completeness of the PX-1 and PX-2 metabolic pathways, it is not only recommended but also necessary to use different assays.

## Introduction

Since their emergence on the drug market in 2004, synthetic cannabinoids (SCs) have been challenging and difficult to detect or to identify for those engaged in toxicological analytics—mainly due to their high chemical diversity, arising from the continuous introduction of new substituents and alterations of the core structures [1–3]. A troubling consequence of SC modifications is an altered affinity to the cannabinoid receptor system [2, 4, 5], which can lead to severe undesired side-effects, such as paranoia, hallucinations, agitation, anxiety, seizures, elevated blood pressure and fatal intoxications [6–8]. Urine samples (a sample matrix of choice due to its abundance and non-invasive sample capture) have proven particularly challenging due to extensive metabolism of SCs. In most cases, only metabolites of SCs can be found in urine samples, making data from metabolism studies vital for target-based urine analysis.

**Fig. 1:**
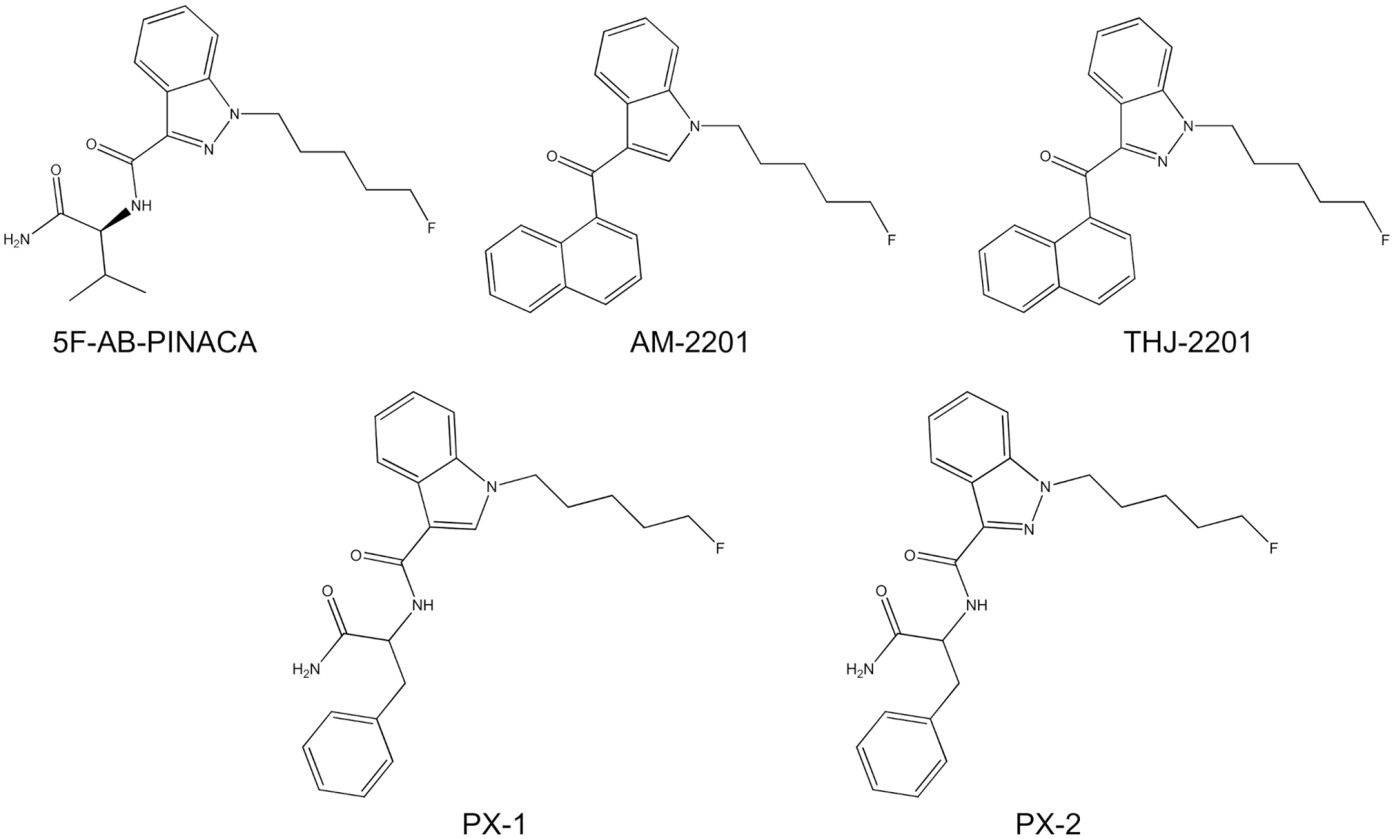
Chemical structures of 5F-AB-PINACA, AM-2201, THJ-2201, PX-1 and PX-2

The studied SC, PX-1 (*N*-(1-amino-1-oxo-3-phenylpropan-2-yl)-1-(5-fluoropentyl)-1*H*-indole-3-carboxamide), also known as SRF-30 and 5F-APP-PICA, and its  indazole analogue PX-2 (*N*-(1-amino-1-oxo-3-phenylpropan-2-yl)-1-(5-fluoropentyl)-1*H*-indazole-3-carboxamide), also known as 5F-APP-PINACA, FU-PX and PPA(N)-2201, were first listed by the European Monitoring Centre for Drugs and Drug Addiction (EMCDDA) on November 25th 2014 and November 6th 2014 in Sweden [9]. Both SCs have a higher affinity to cannabinoid receptor 2 (CB_2_) (Ki: PX-1 164 ± 17 nM, PX-2 58 ± 17 nM) than to CB_1_ (Ki: PX-1 485 ± 117 nM, PX-2 127 ± 43 nM) [10]. Both substances showed structural similarities to 5F-AB-PINACA, THJ-2201 and AM-2201 (see Fig. 1). PX-1 and AM-2201 share an indole core and 5-flouropentyl tail, and 5F-AB-PINACA shares the carboxamide linker with PX-1 and PX-2. THJ-2201 and 5F-AB-PINIACA also share their 5-fluropentyl indazole core with PX-2.

In vitro metabolisms of THJ-2201, 5F-AB-PINACA and AM-2201 have been extensively studied. To date, in vitro studies on the metabolism of PX-1, applying human hepatocyte assays and pooled human liver microsomes (pHLM), have been published by Cooman et al. [11], Presley et al. [12] and Fabregat-Safont et al. [13]. Their findings showed an SC’s typical metabolism. Only Cooman et al. [11] investigated PX-2 while Fabregat-Safont et al. [13] studied also the phase II metabolism of PX-1. The number of phase I metabolites which they found ranged from six to ten. They all found monohydroxylation of PX-1 and PX-2 and also further hydroxylations, such as dihydroxylation, amide hydrolysis, carboxylation and dealkylation [11–13]; monohydroxylation, oxidative defluorination, and defluorination with subsequent carboxylation [14–24] were the most dominant alterations. Dihydrodiol metabolites, detected for 5F-AB-PINACA and THJ-2201 were not observed for PX-1 and PX-2 [14, 17, 18].

Metabolism research on SCs is mostly conducted via in vitro studies prior to analysis of urine samples from SC users. Current in vitro metabolism assays in use are: pHLM; selected cytochrome P450 (CYP) monooxygenase isoforms; human hepatocytes; and, for in vivo studies, animal models (e. g., rat and mice) as well as authentic human urine samples. All of these methods are suitable for establishing a library of metabolites that can be expected in authentic urine samples [14, 16, 18, 24–29]. A further in vitro study made use of filamentous fungi, such as *Cunninghamella elegans* (*C. elegans*), due to the similarity displayed by the CYP enzyme system to the mammalian enzyme system. *C. elegans* has been used in metabolism testing of several pharmaceuticals and some SCs [23, 30–35]. The aim of this study is to fill the information gaps on the metabolisms of PX-1 and PX-2 (including involved CYP isoforms) and to compare the findings with the metabolisms of structurally similar SCs. A further aim is to add to current knowledge on *C. elegans*-based SC metabolism data. To compare fungal biotransformation data, the more common pHLM incubation and eight different CYPs were used. These CYPs were chosen based on SC metabolism data [36].

## Materials and methods

### Chemicals and reagents

Solid reference materials of PX-1 and PX-2 with a purity of ≥ 98.0% were purchased from Cayman Chemical (Ann Arbor, MI, USA). Working solutions of both PX-1 and PX-2 (1 mg/mL) were prepared in dimethyl sulfoxide (DMSO). For fungal incubation, 1.3 mg of PX-1 and 1.4 mg PX-2 were prepared in 200 µL DMSO, leading to a concentration of 6.5 mg/mL PX-1 and 7 mg/mL PX-2.

Pro-analysis grade sodium sulphate (Na_2_SO_4_), sodium chloride (NaCl), potassium citrate (hydrate), and liquid chromatography–mass spectrometry (LC–MS) grade ammonium formate and formic acid were obtained from Merck (Darmstadt, Germany). LC–MS grade water and acetonitrile (ACN), high-performance liquid chromatography (HPLC) gradient grade water, ACN, methanol (MeOH), dichloromethane (DCM), DMSO, and 2-propanol were purchased from Carl Roth (Karlsruhe, Germany). Gentest™ reduced β-nicotinamide adenine dinucleotide phosphate (NADPH) regenerating system solution A (26 mM β-nicotinamide adenine dinucleotide phosphate (NADP^+^), 66 mM MgCl_2_ in water, 66 mM d-glucose-6-phosphate) and solution B (40 U/mL  glucose-6-phosphate dehydrogenase (Glc-6-P-DH; EC 1.1.1.49) in sodium citrate) were purchased from Corning (Woburn, MA, USA). Superoxide dismutase (SOD) (EC 1.15.1.1; from bovine erythrocytes), with a specific activity of 3 kU/mg protein, were purchased from Merck.

Corning supplied pHLM from a 35-donor pool (protein content of 20 mg/mL, and total CYP content of 300 pmol/mg). The following seven “Easy Cyp” (concentration of 1 nmol/mL) were supplied by Cypex (Dundee, Scotland, UK) via Tebu-bio (Offenbach, Germany): CYP1A2, CYP2B6, CYP2C9, CYP2C19, CYP2D6, CYP3A4 and CYP3A5. CYP2B6, CYP2C9, and CYP3A5 (preparations also contained CYP reductase) were supplied by Cypex via Tebu-bio (Offenbach, Germany). A suspension of viable cells of *C. elegans* (ATCC^®^ 10028b™) in cryoprotectant was obtained from ATCC (Manassas, VA, USA). Potato dextrose agar, peptone (enzymatic digest from soybeans), yeast extract, malt extract and d-glucose (monohydrate) were obtained from Fluka (Sigma-Aldrich, St. Louis, MO, USA). A 500 mL volume of potato dextrose agar were prepared by dissolving 18.5 g agar in deionized water; 500 mL of growth media were prepared by dissolving 2.5 g peptone, 1.5 g of yeast extract, 1.5 g of malt extract and 7.5 g d-glucose in deionized water.

### Microsomal incubation with pHLM and CYPs

Microsomal incubation was carried out based on previously published metabolism studies [27, 37] and on manufacturer’s guidelines. Microsomal incubation time was varied between 1 and 4 h, during optimisation. Optimal incubation time, delivering the highest metabolite abundancies, was established to be 3 h, with a 4 h incubation time showing no additional metabolites. The following incubations were performed, therefore, at 3 h incubation time: 177.6 µL 100 mM phosphate buffer (pH 7.4), 12.5 µL NADPH generating system solution A, 10.0 µL NADPH generating system solution B and 20.0 µL SOD (240 U/mL final activity) were mixed with 10.0 µL of  SC working solution (1 mg/mL in ACN/DMSO = 80:10, v/v), respectively; 10.0 µL ACN/DMSO = 80:10 (v/v) for blank. After vortexing, 20.0 µL pHLM (1.6 mg/mL final concentration) or 20 µL of CYPs (final concentration 80 pmol/mL each) were added to start the incubation process. For the control, 20.0 µL phosphate buffer were added. Final concentrations were: 101.14 µM of PX-1 and 100.88 µM for PX-2. Samples were vortexed and incubated for 3 h at 37 °C and shaken at 10 Hz using a Thermomixer 5436 from Eppendorf (Hamburg, Germany). Vial slots of the Thermomixer were filled with 100 µL water for improved thermal conduction. Quenching was carried out by adding 250 µL ice-cold ACN followed by vortexing and 10 min. storage at –20 °C. Quenched samples were centrifuged for 10 min at 4 °C/9000 *g* in a Heraeus Fresco 17 centrifuge (Thermo Scientific, Waltham, MA, USA). For clean-up measurements, a manual solid-phase extraction (SPE) was carried out.

### *Cunninghamella elegans* biotransformation

*Cunninghamella elegans* was cultivated, prior to fungal incubation, according to the process described by Watanabe et al*.* [23]. *C. elegans* cells were cultivated on potato dextrose agar, sterilized at 120 °C for 20 min in an autoclave (model 3150EL; Systec, Linden, Germany) and filled into sterile Petri dishes (Becton  Dickinson, Franklin Lakes, NJ, USA). Viable *C. elegans* cells were plated out onto cooled agar using a sterile inoculation loop. Mycelia were allowed to grow for five days at 25 °C using a heat cabinet (model ED 240; Binder, Tuttlingen, Germany). Growth medium, baffled flasks, cotton stoppers and physiological saline solution (0.15 M) were also sterilized at 120 °C for 20 min, using the previously described autoclave. After 5 days of growth, mycelia from five plates were harvested and pooled in 35 mL of sterile physiological saline solution. Three baffled flasks were filled with 100 mL sterile growth media. Two of the three flasks were inoculated with 3 mL of mycelia solution, while the third flask was used as a negative control. All flasks were sealed with cotton stoppers and incubated for 3 days at 27 °C and shaken at 3 Hz using an incubator (model 3032; GFL GmbH, Burgwedel, Germany). Following fungal incubation, 200 µL PX-1 (6.5 mg/mL) and PX-2 (7.0 mg/mL) solution were added to fungal-infested growth media, which corresponded to 32.87 mM PX-1 and 35.31 mM PX-2, respectively. A 200-µL aliquot of DMSO was added to the control. They were incubated for 6 days, followed by suction filtration with a Buchner funnel. Filtrated media were extracted three times with 50 mL DCM, leading to a 150 mL organic layer. The organic layer was washed three times with 50 mL deionized water, and the organic layer was collected and subsequently dried with Na_2_SO_4_. Extracts were evaporated to dryness at ambient pressure and 60 °C, using a rotary evaporator (model VV200) equipped with a water bath (model WB200) (Heidolph, Kelheim, Germany), and then dissolved in 500 µL ACN/deionized water (50:50, v/v).

### Solid-phase extraction

The SPE of the incubation samples of pHLM, CYP, and fungal liquid-liquid extracts were manually conducted using a VacMasert™ 10 vacuum manifold (Biotage, Uppsala, Sweden) to clean up and concentrate PX-1, PX-2 and their metabolites. Conditioning of SPE cartridges (Empore™ Disk C18, 3M™; Agilent Technologies, Santa Clara, CA, USA) was achieved using 2 mL each of MeOH and deionized water. The supernatant from microsomal incubation samples (450 µL) was diluted with 2500 µL of deionized water. A 500 µL volume of fungal incubation solution were diluted with 2500 µL deionized water. After sample loading, the cartridges were washed two times with 2 mL deionized water and dried for at least 1 min under vacuum (− 850 kPa). Analytes were eluted by loading 1 mL ACN four times onto the cartridges. All steps were performed at a maximum vacuum of − 500 kPa. Collected eluates were evaporated to dryness under a gentle nitrogen stream at room temperature using a Vapotherm evaporator (Barkley, Leopoldshöhe, Germany). Dried residues were dissolved in 100 µL solution of 2 mM ammonium formate buffer with 0.1% formic acid and ACN with 0.1% formic acid (60:40, v/v) and transferred into HPLC vials with inserts.

### Liquid chromatography–high-resolution mass spectrometry conditions

Liquid chromatography–high-resolution mass spectrometry (LC–HRMS) experiments were conducted on a Vanquish UHPLC system (Thermo Fisher Scientific, Bremen, Germany) coupled to a Thermo Scientific™ Q Exactive™ HF-X Fourier transformation mass spectrometer (FTMS) from Thermo Fisher Scientific, equipped with a heated electrospray ionization (HESI-II) source and a hybrid-quadrupole-orbitrap. Chromatographic separation was performed at ambient temperature on an Agilent Poroshell 120 Vanquish ultra-high-performance liquid chromatography (UHPLC), with a column EC-C8 (2.7 µm, 3.0 × 50 mm) from Agilent Technologies.

As mobile phases, solutions of 0.1% formic acid in both water (solvent A) and ACN (solvent B) were used. Initial conditions were 1% of solvent B, increasing over 10 min to 40% and then to 99% within 0.9 min. After 0.1 min run time, the system was returned to initial conditions and held for a further 3 min. Total run time was 14 min. The flow rate was set to 0.4 mL/min and the autosampler temperature was set to 10 °C. The injection volume was 3 μL.

The mass spectrometer was operated in positive ionization mode with an ion spray voltage  at 4000 V and a capillary temperature  at 320 °C. Nitrogen, provided by a nitrogen generator from CMC Instruments (Eschborn, Germany), was used as the collision gas and for all ionization assisting gases. The HRMS system was calibrated using the manufacturer`s recommendations, enabling mass errors < 5 ppm. Full MS data were collected over a scan range of *m/z* 150 to 2000 and at a resolution of 60,000 full width at half maximum (FWHM). Up to five targeted MS/MS experiments were conducted simultaneously in the product ion monitoring mode. Targeted MS/MS data were acquired at a resolution of 30,000 FWHM. The automatic gain control target was set to 3e6 for full MS and 5e5 for product ion scan. For targeted MS/MS experiments, the isolation window of the quadrupole was set to *m/z* 1.5. Metabolites and corresponding product ions were predicted using the ChemSketch software (2017.1.2 version; ACD Labs, Toronto, Canada) mass spectrometry scissors tool. HRMS results were then compared with the predicted metabolites.

## Results

PX-1 metabolites found after pHLM incubation are listed in Table 1 and PX-2 metabolites in Table 2, including biotransformation, retention time, elemental composition, accurate mass, mass error and diagnostic product ions. Main metabolites were determined by dividing the raw peak areas of individual metabolites by the peak area of the most abundant metabolite identified. These are ranked in Tables 1 and 2.

**Table 1:** Metabolite profiling for PX-1 with pooled human liver microsomes (pHLM): retention times, accurate masses of precursor ions (*m/z*), elemental compositions, diagnostic product ions, and mass errors, and ranks were determined by dividing the raw peak areas of individual metabolites by the peak area of the most abundant metabolite identified

ID	Precursor ion [*m/z*]	Modification	Position	Elemental composition [+ H]	Rt [min]	Product ion [*m/z*]	Mass error [ppm]	Rank
M1	412.20	Monohydroxylation	P	C_23_H_27_FN_3_O_3_	10.11	395.18, 248.11, 144.04	0.59	19
M2	412.20	Monohydroxylation	I	C_23_H_27_FN_3_O_3_	10.41	395.18, 248.11, 160.04	0.37	10
M3	412.20	Monohydroxylation	P	C_23_H_27_FN_3_O_3_	10.6	395.18, 248.11, 144.04	0.67	17
M4	412.20	Monohydroxylation	P	C_23_H_27_FN_3_O_3_	10.65	395.18, 248.11, 144.04	0.94	11
M5	412.20	Monohydroxylation	I	C_23_H_27_FN_3_O_3_	11.09	395.18, 248.11, 160.04	1.35	25
M6	412.20	Monohydroxylation	P or I	C_23_H_27_FN_3_O_3_	11.25	248.11, 158.06	1.85	24
M7	412.20	Monohydroxylation	B	C_23_H_27_FN_3_O_3_	11.43	395.18, 289.13, 232.11, 163.09	0.23	8
M8	412.20	Monohydroxylation	B	C_23_H_27_FN_3_O_3_	11.48	395.18, 289.13, 232.11, 163.09	0.25	6
M9	394.21	Defluorination and monohydroxylation	P	C_23_H_28_N_3_O_3_	10.18	377.19, 230.12, 144.04	− 0.72	1
M10	394.21	Defluorination and monohydroxylation	P	C_23_H_28_N_3_O_4_	11.01	230.12, 144.04	− 3.04	5
M11	324.13	Dealkylation and monohydroxylation	I	C_18_H_18_N_3_O_3_	7.26	307.11, 279.11, 160.04	1.09	20
M12	324.13	Dealkylation and monohydroxylation	I	C_18_H_18_N_3_O_3_	7.86	307.11, 279.11, 201.07, 160.04	− 0.10	27
M13	408.18	Carboxypentyl	P	C_23_H_26_N_3_O_4_	10.19	391.17, 244.10, 144.04	− 0.66	7
M14	392.19	Defluorination and ketone formation	P	C_23_H_26_N_3_O_3_	10.44	375.17, 228.10	0.68	12
M15	392.19	Defluorination and ketone formation	P	C_23_H_26_N_3_O_3_	10.64	246.09, 228.10, 210.09, 144.04	0.82	21
M16	410.19	Defluorination and dihydroxylation	P and I	C_23_H_28_N_3_O_4_	8.26	393.18, 291.17, 264.10, 246.11, 160.04	0.33	22
M17	410.19	Defluorination and dihydroxylation	I;I or P;I	C_23_H_28_N_3_O_4_	8.71	393.18, 264.10, 246.11, 158.06	0.53	28
M18	410.19	Defluorination and dihydroxylation	P and I	C_23_H_28_N_3_O_4_	8.87	393.18, 246.11, 160.04	0.15	29
M19	410.19	Defluorination and dihydroxylation	P and B	C_23_H_28_N_3_O_4_	9.13	393.18, 230.12, 163.09	− 0.03	9
M20	410.19	Defluorination and dihydroxylation	P and B	C_23_H_28_N_3_O_4_	9.34	393.18, 230.12, 163.09, 144.04	0.36	14
M21	397.10	Distal amide hydrolysis		C_23_H_26_FN_2_O_3_	11.79	232.11, 144.04	− 0.57	2
M22	413.19	Distal amide hydrolysis and monohydroxylation	P	C_23_H_26_FN_2_O_4_	10.10	396.18, 248.11, 144.04, 105.07	− 0.30	23
M23	413.19	Distal amide hydrolysis and monohydroxylation	P	C_23_H_26_FN_2_O_4_	10.98	248.11, 144.04	0.86	26
M24	413.20	Distal amide hydrolysis and monohydroxylation	I	C_23_H_26_FN_2_O_4_	11.09	396.18, 248.11, 160.04	1.34	30
M25	413.20	Distal amide hydrolysis and monohydroxylation	P or I	C_23_H_26_FN_2_O_4_	11.23	248.11, 158.06	1.49	15
M26	413.20	Distal amide hydrolysis and monohydroxylation	P or I	C_23_H_26_FN_2_O_4_	11.29	248.11, 158.06	0.95	18
M27	413.20	Distal amide hydrolysis and monohydroxylation	B	C_23_H_26_FN_2_O_4_	11.41	378.2, 289,13, 232.11, 163.09	1.29	16
M28	413.20	Distal amide hydrolysis and monohydroxylation	B	C_23_H_26_FN_2_O_4_	11.48	396.18, 289.13, 232.11, 163.09	1.03	13
M29	395.19	Distal amide hydrolysis, defluorination and monohydroxylation	P	C_23_H_27_N_2_O_4_	11.03	230.12, 144.04	− 0.51	3
M30	308.13	*N*-Desalkylation		C_18_H_18_N_3_O_2_	9.19	291.11, 263.12, 165.10, 144.04	− 1.53	4
PX-1	396.20	Parent compound		C_23_H_27_FN_3_O_2_	11.68	379.20, 232.11, 144.05	− 1.03	

*P* pentyl, *I* indole, *B* benzyl, *Rt* retention time

**Table 2:** Metabolite profiling for PX-2 with pHLM: retention times, accurate mass precursor ions (*m/z*), elemental compositions, diagnostic product ions, and mass errors, and  ranks were determined by dividing the raw peak areas of individual metabolites by the peak area of the most abundant metabolite identified

ID	Precursor ion [*m/z*]	Modification	Position	Elemental composition [+ H]	Rt [min]	Product ion [*m/z*]	Mass error [ppm]	Rank
F1	413.19	Monohydroxylation	P or I	C_22_H_26_FN_4_O_3_	10.45	368.18, 267.11, 249.10	0.78	14
F2	413.19	Monohydroxylation	P	C_22_H_26_FN_4_O_3_	11.28	368.18, 267.11, 249.10, 175.05, 145.04	− 1.51	12
F3	413.19	Monohydroxylation	P	C_22_H_26_FN_4_O_3_	11.41	368.18, 267.11, 249.10, 145.04	0.56	10
F4	395.20	Defluorination and monohydroxylation	P	C_22_H_27_N_4_O_3_	11.27	378.18, 350.19, 231.11 213.10, 145.04	0.99	2
F5	409.18	Carboxypentyl	P	C_22_H_24_N_4_O_4_	10.32	364.17, 245.09, 217.10	0.55	4
F6	411.20	Defluorination and dihydroxylation	P;P	C_22_H_27_N_4_O_4_	8.74	366.18, 247.11, 175.05, 145.04	0.04	15
F7	411.20	Ketone	P	C_22_H_27_N_4_O_4_	8.88	366.18, 247.11, 229.10, 191.05	− 1.33	13
F8	411.20	Ketone	P	C_22_H_27_N_4_O_4_	9.70	366.18, 247.11, 229.10, 175.05, 145.04	0.54	11
F9	398.19	Distal amide hydrolysis		C_22_H_25_FN_3_O_3_	11.88	251.12, 233.11, 213.10, 145.04	− 0.63	1
F10	414.18	Distal amide hydrolysis and monohydroxylation	P	C_22_H_25_FN_3_O_4_	11.28	414.18, 396.17, 368.18, 267.11, 249.10, 145.04	− 0.12	3
F11	414.18	Distal amide hydrolysis and monohydroxylation	P	C_22_H_25_FN_3_O_4_	11.40	414.18, 396.17, 368.18, 267.11, 249.10, 145.04	0.99	5
F12	414.18	Distal amide hydrolysis and monohydroxylation	P	C_22_H_25_FN_4_O_3_	11.48	414.18, 396.17, 368.18, 350.17, 267.11, 249.10, 145.04	1.65	9
F13	414.18	Distal amide hydrolysis and monohydroxylation	B	C_22_H_25_FN_4_O_3_	11.52	414.18, 396.17, 368.18, 267.11, 233.11, 213.10, 165.05	1.72	7
F14	414.18	Distal amide hydrolysis and monohydroxylation	I	C_22_H_25_FN_3_O_4_	11.61	396.17, 368.18, 267.11, 249.10, 161.03	1.83	8
F15	414.18	Distal amide hydrolysis and monohydroxylation	I	C_22_H_25_FN_3_O_4_	11.66	368.18, 267.11, 249.10, 161.03	0.54	6
PX-2	397.19	Parent compound		C_22_H_26_FN_4_O_2_	11.7	352.18, 251.12, 233.11, 213.10, 145.04	0.92	

### Metabolic profile of PX-1

The PX-1 peak area dropped to 71% after 1 h and 37% after 3 h of pHLM incubation. Of the tested incubation times, 3 h incubation revealed the best result in terms of number of metabolites and abundancy. After incubation with pHLM for 3 h, PX-1 yielded 30 metabolites (M1–M30, listed in Table 1), belonging to eight different groups. Metabolites eluted at 7.26–11.79 min, while the parent drug eluted at 11.68 min. Metabolites were generated by monohydroxylation, oxidative defluorination, dealkylation, dealkylation with monohydroxylation, carboxamide oxidation, defluorination with dihydroxylation, defluorination in combination with ketone formation and carboxamide oxidation and oxidative defluorination to carboxylic acid.

**Fig. 2:**
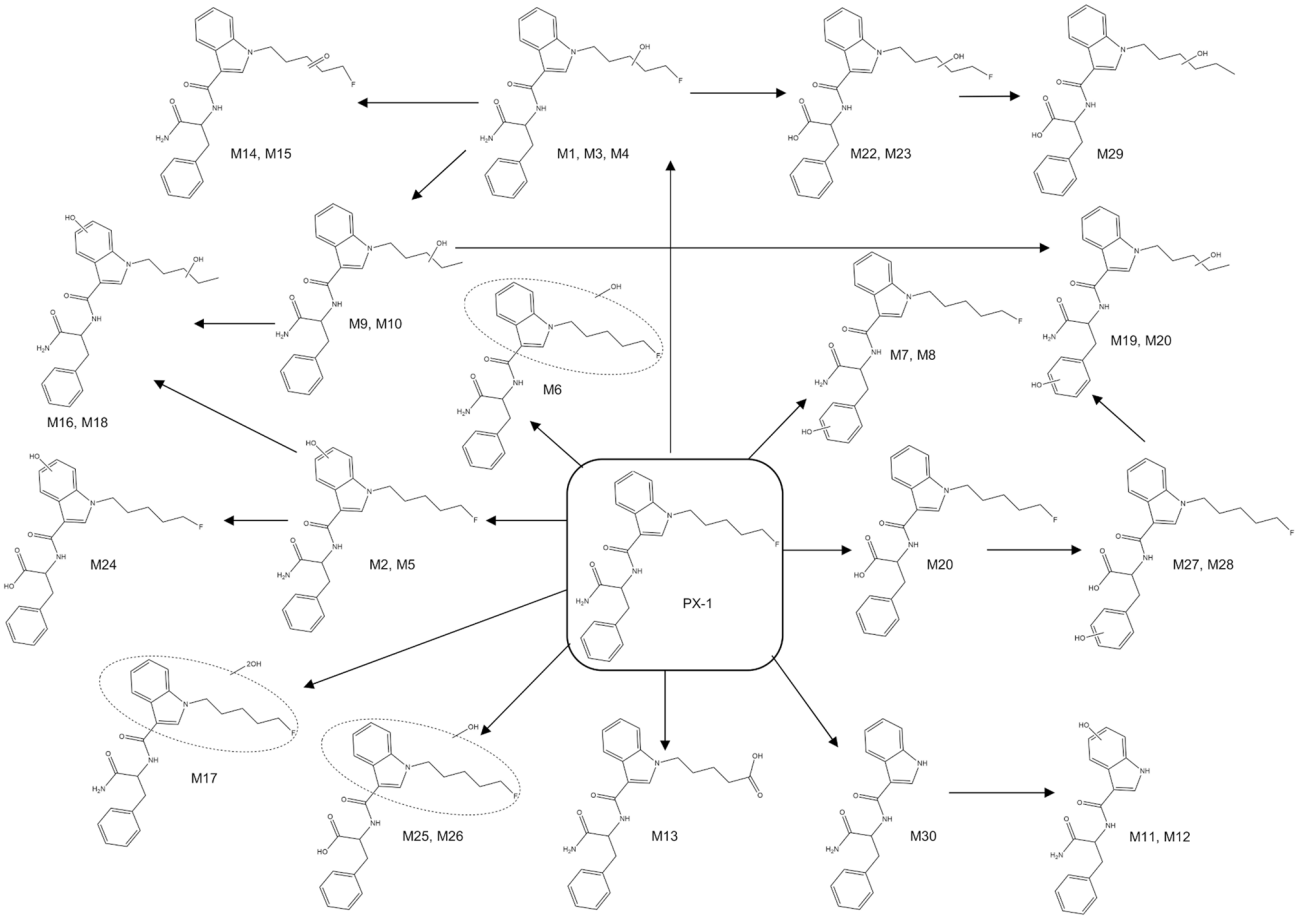
Proposed metabolic pathways of PX-1

Fungal incubation of PX-1 showed a total of 16 metabolites, and a total of 26 metabolites were observed in PX-1 incubation experiments with CYP isoforms. All these metabolites were identical to those observed from pHLM incubation (Table 3). An overview of metabolite structures and the predicted metabolic pathways of PX-1 is shown in Fig. 2. In addition, six further metabolites (MU1–MU6) were observed, for which the position of the metabolic modification could not revealed with confidence.

**Table 3:** Comparison of the three in vitro models of PX-1 metabolism (pHLM, CYPs and fungus)

ID	Precursor ion [*m/z*]	Modification	Position	Rt [min]	pHLM	1A2	3A4	3A5	2B6	2C9	2C19	2D6	2E1	*C. elegans*
M1	412.20	Monohydroxylation	P	10.11	x	x	–	x	x	x	x	x	x	x
M2	412.20	Monohydroxylation	I	10.41	x	–	–	–	x	–	–	–	x	x
M3	412.20	Monohydroxylation	P	10.6	x	x	–	–	x	–	–	–	–	–
M4	412.20	Monohydroxylation	P	10.65	x	–	–	x	x	x	x	–	x	x
M5	412.20	Monohydroxylation	I	11.09	x	x	–	x	x	x	x	x	x	-
M6	412.20	Monohydroxylation	P or I	11.25	x	x	–	–	–	–	–	–	–	x
M7	412.20	Monohydroxylation	B	11.43	x	x	–	x	x	x	–	–	x	x
M8	412.20	Monohydroxylation	B	11.48	x	x	–	x	x	x	–	–	x	x
M9	394.21	Defluorination and monohydroxylation	P	10.18	x	–	x	x	x	x	x	x	x	x
M10	394.21	Defluorination and monohydroxylation	P	11.01	x	x	–	–	–	–	–	–	–	–
M11	324.13	Dealkylation and monohydroxylation	I	7.26	x	–	–	–	x	–	x	–	x	–
M12	324.13	Dealkylation and monohydroxylation	I	7.86	x	–	–	–	–	–	–	–	–	–
M13	408.18	Carboxypentyl	P	10.19	x	x	x	x	x	x	x	–	x	x
M14	392.19	Defluorination and ketone formation	P	10.44	x	x	–	–	–	–	–	–	–	–
M15	392.19	Defluorination and ketone formation	P	10.64	x	x	–	–	–	–	–	–	–	x
M16	410.19	Defluorination and dihydroxylation	P and I	8.26	x	–	–	–	–	–	–	–	–	–
M17	410.19	Defluorination and dihydroxylation	I;I or P;I	8.71	x	–	–	–	–	–	x	x	–	x
M18	410.19	Defluorination and dihydroxylation	P and I	8.87	x	–	x	–	–	–	–	–	–	–
M19	410.19	Defluorination and dihydroxylation	P and B	9.13	x	–	x	–	x	–	–	–	x	–
M20	410.19	Defluorination and dihydroxylation	P and B	9.34	x	–	–	–	x	–	–	–	–	–
M21	397.10	Distal amide hydrolysis		11.79	x	x	–	x	x	x	x	x	x	x
M22	413.19	Distal amide hydrolysis and monohydroxylation	P	10.10	x	–	–	–	x	–	x	x	x	x
M23	413.19	Distal amide hydrolysis and monohydroxylation	P	10.98	x	–	–	–	–	–	–	–	–	x
M24	413.20	Distal amide hydrolysis and monohydroxylation	I	11.09	x	–	–	–	x	–	–	–	x	–
M25	413.20	Distal amide hydrolysis and monohydroxylation	P or I	11.23	x	–	–	–	–	–	–	–	–	x
M26	413.20	Distal amide hydrolysis and monohydroxylation	P or I	11.29	x	–	–	–	–	–	–	–	–	–
M27	413.20	Distal amide hydrolysis and monohydroxylation	B	11.41	x	–	–	–	x	x	–	–	–	x
M28	413.20	Distal amide hydrolysis and monohydroxylation	B	11.48	x	–	–	–	x	x	–	–	–	–
M29	395.19	Distal amide hydrolysis and defluorination and monohydroxylation	P	11.03	x	x	–	–	–	–	–	–	–	x
M30	308.13	*N*-Desalkylation		9.19	x	x	x	x	x	x	–	–	x	–
PX-1	396.20	Parent compound		11.68	x	x	x	x	x	x	x	x	x	x

*pHLM* pooled human liver microsomes, *CYPs* cytochrome p450s

### Characteristic product ions of PX-1

**Fig. 3:**
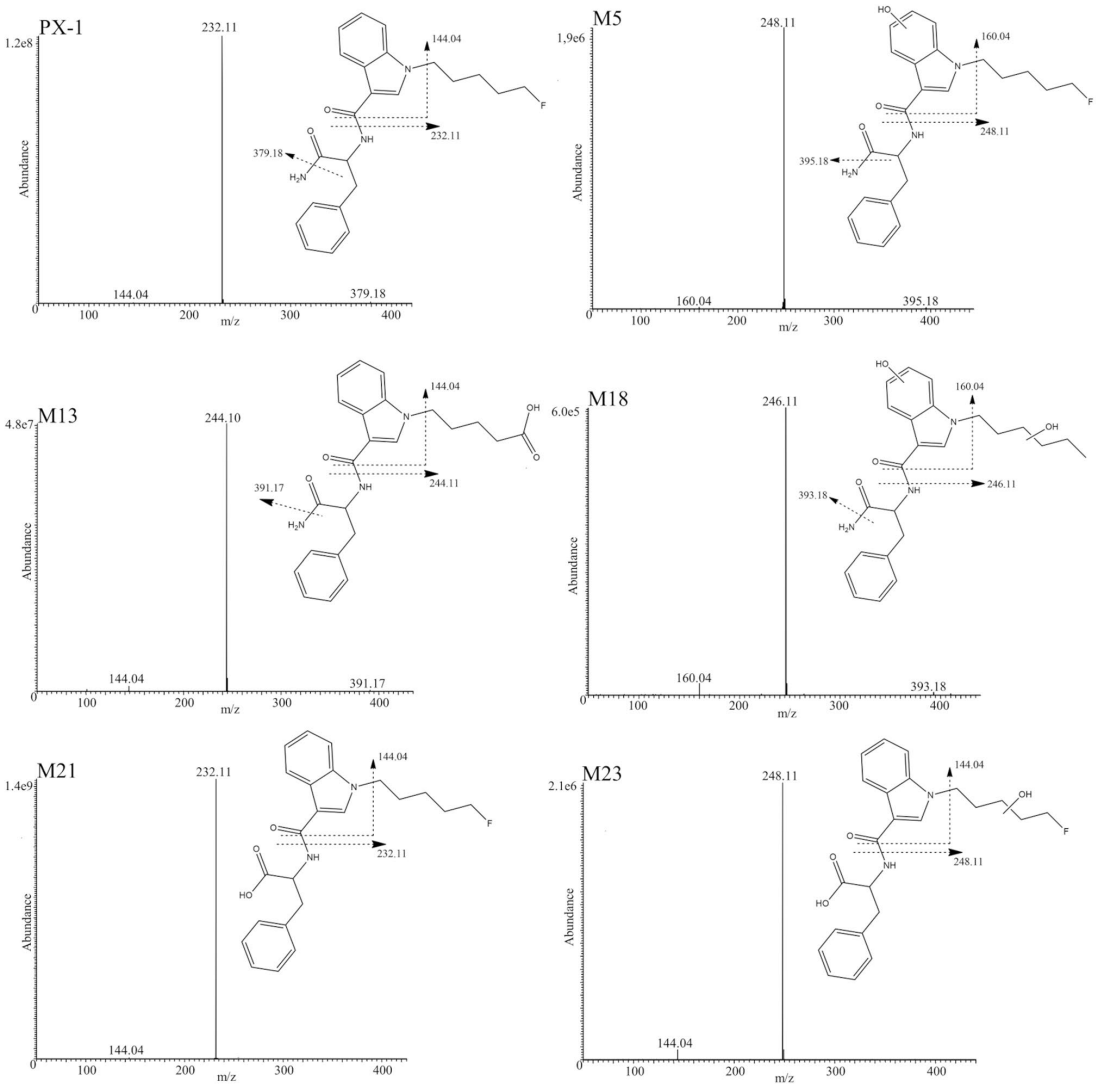
Product ion spectra of PX-1 and its five metabolites with proposed structures and fragmentation patterns

The product ion mass spectrum of PX-1 showed three characteristic product ions (Fig. 3) at *m/z* 232.11 (unchanged pentylindole acylium ion), 379.18 (removal of amine group) and 144.04 (unaltered indole acylium ion).

### Hydroxylated metabolites of PX-1

Oxidation of the parent compound occurred at different positions. Eight monohydroxylated metabolites were found (M1–M8). Three of those were monohydroxylated at the fluoropentyl side chain (M1, M3, M4). The position of the alteration is indicated by the observed product ions *m/z* 248.11 (monohydroxylated indoleacylium ion with fluoropentyl side chain) and *m/z* 144.04 (unaltered indole acylium ion without side chain) as the most abundant fragments. M2 and M5 showed the product ions at *m/z* 248.11 and 160.04 (monohydroxylated indole acylium ion), which indicate monohydroxylations at the indole moiety. Metabolite M6 showed the product ion at *m/z* 158.06, which is considered a methylindole acylium ion [13]. The presence of the product ion *m/z* 248.11 indicates monohydroxylation at the indole or pentyl moiety. The metabolites M7 and M8 were identified as monohydroxylations at the benzyl moiety by detection of *m/z* 144.04, 232.11 (indole acylium ion with fluoropentyl side chain) and 289.13 (molecule fragment without monohydroxybenzyl-moiety and the distal amide) [12]. The product ions at *m/z* 230.12 (*N*-pentyl indole acylium ion) and 144.04 indicate the monohydroxylation of the pentyl side chain after defluorination (M9 and M10). The metabolites M11 and M12 represent dealkylated monohydroxylated metabolites. The presence of the product ion at *m/z* 160.04 indicated monohydroxylation at the indole moiety for M11 and M12. For those metabolites, the observed product ion at *m/z* 307.11 is considered to be the result of dealkylation in combination with an in-source elimination of NH_3_.

Further oxidations such as dihydroxylation, ketone formation and carboxylation, were observed. The observed metabolite M13, identified by *m/z* 244.10 and 144.04, appeared to be a result of oxidative defluorination to carboxylic acid, for which no hydroxylation reaction at the indole core (M13) was observed. Oxidative defluorination to carboxylic acid is well known for the most fluorinated synthetic cannabinoids. Metabolites M14 and M15 showed the characteristic product ions at *m/z* 228.10 and 144.04 for defluorinated pentyl side chain with ketone formation. Five dihydroxylated and defluorinated metabolites (M16–M20) were detected. The product ions *m/z* 230.12, 163.09, and 144.04 together with the absence of *m/z* 160.04 are assumed to arise from hydroxylation of the benzyl moiety and hydroxylation of the pentyl side chain (M19 and M20). The presence of the product ions *m/z* 160.04 and 246.11 are considered to be due to pentyl side chain hydroxylation and indole hydroxylation (M16 and M18). Similar to M6, product ion *m/z* 158.06 was also observed for metabolite M17. With the presence of *m/z* 246.11, M17 is considered to be a dihydroxylated metabolite, but the hydroxylated sites could not be established.

### Metabolites generated by amide hydrolysis of PX-1

Terminal amide hydrolysis resulted in the most abundant metabolite (M21) of all the PX-1 metabolites. This has previously been observed for 5F-AB-PINACA [14]. M21 showed all the common fragments observed for PX-1 and eluted 0.1 min after it. Further metabolites detected with amide hydrolysis yielded a total of seven monohydroxylated metabolites, and one defluorinated as well as monohydroxylated metabolite. Metabolites (M22–M28) were monohydroxylated, M22 and M23 of which were oxidated at the fluoropentyl side chain, with the product ions at *m/z* 248.11 and 144.04 indicating no oxidation at the indole core. The product ions observed for M24 indicated oxidation at the indole core by the presence of *m/z* 160.04 and 248.11 along with the absence of *m/z* 144.04. Metabolites M27 and M28 are considered to be monohydroxylated at the benzyl ring of the secondary moiety, indicated by the observation of *m/z* 289.13 and 163.09, and the product ion *m/z* 232.11 (unchanged pentylindole acylium ion). M25 and M26 showed the product ion *m/z* 248.11, which indicated  a monohydroxylation either at the fluoropentyl side chain or at the indole core. Defluorination with amide hydrolysis and subsequent monohydroxylation resulted in M29 with *m/z* 230.12 and 144.04.

### Dealkylated metabolite of PX-1

M30 is considered as a dealkylated metabolite, indicated by the observed fragments *m/z* 291.11, 263.12 and 144.04.

### Unidentified metabolites (MU) of PX-1

In addition to the 30 metabolites identified, 6 further metabolites (MU1–MU6) were detected for which it was not possible to identify the modification and/or location of the metabolic modification with certainty. MU1 ([MH^+^] *m/z* 324.13, retention time (Rt) = 8.14 min) could be a dealkylated and monohydroxylated metabolite. MU2 ([MH^+^] *m/z* 410.19, Rt = 8.36 min), MU3 ([MH^+^] *m/z* 410.19, Rt = 8.82 min), and MU4 ([MH^+^] *m/z* 410.19, Rt = 10.5 min) indicated further defluorinated and dihydroxylated metabolites. The ion at *m*/z 410.19 would also lead to a ketone formation at the fluoropentyl side chain. The two metabolites MU5 ([MH^+^] *m/z* 413.19, Rt = 10.4 min) and MU6 ([MH^+^] *m/z* 413.19, Rt = 10.7 min) are thought to be monohydroxylated metabolites, which  were also hydrolyzed at the distal amide. Metabolites MU1, MU2 and MU4 could not be detected in the experiments with CYP isoforms but were detected with fungal incubation. MU5 and MU6 could be detected in the several CYP incubations, but were not found after fungal incubation. MU3 could be detected in the fungal incubations and  was present in several CYP incubations.

### Metabolic pattern of PX-1 after incubation with CYP isoforms and with *C. elegans*

**Fig. 4:**
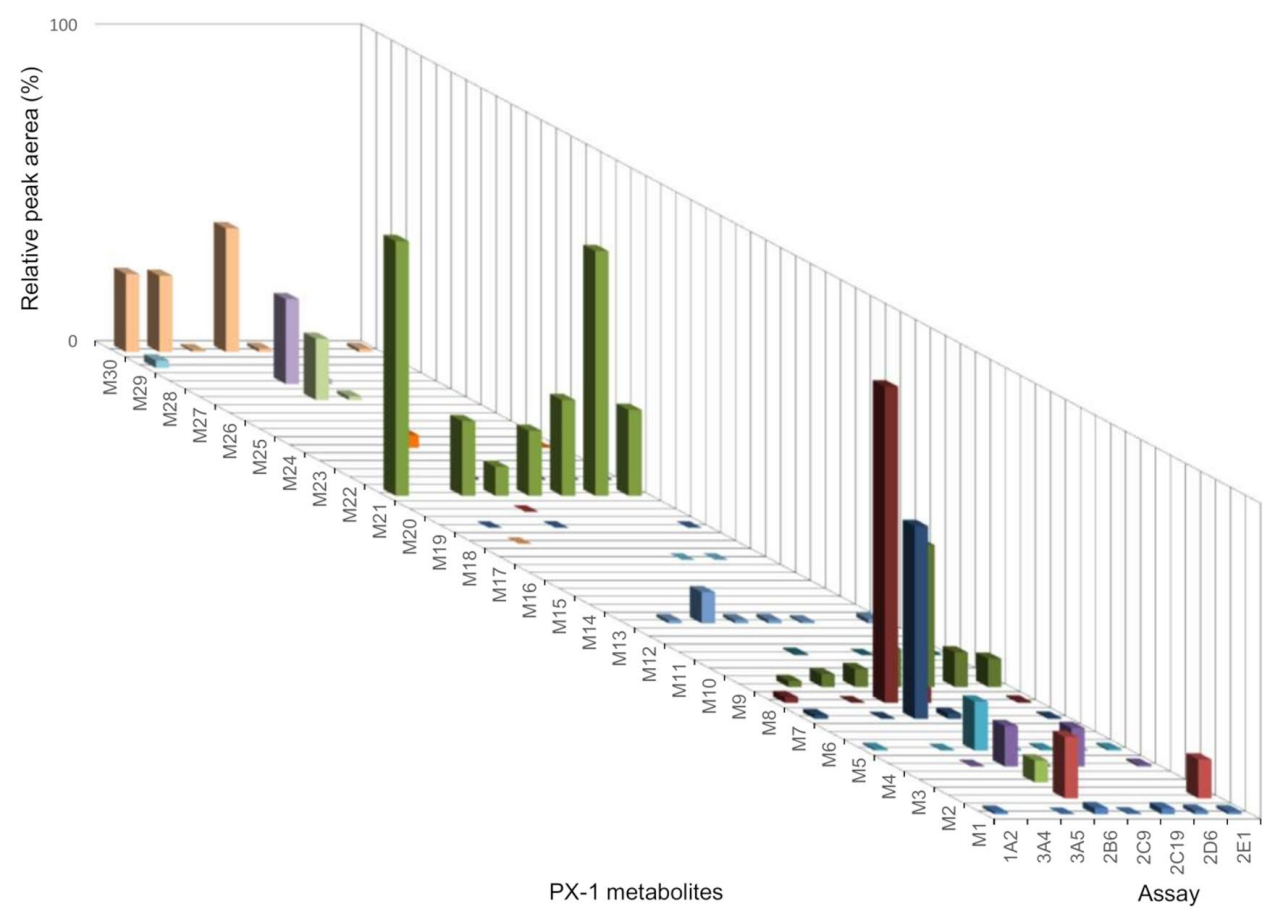
Results of the PX-1 cytochrome P450 (CYP) incubation. *X*-axis shows the different CYP isoforms, *z*-axis shows the metabolites and the *y*-axis the relative peak areas (%). The relative peak areas represent the measured peak areas normalised to the most abundant metabolite identified

For PX-1, no new or CYP- or *C. elegans-*exclusive metabolites could be found. CYP2B6 and CYP2E1 formed 18 and 14 metabolites, respectively, which are similar to pHLM incubation. CYP2B6 and CYP2E1gave the most metabolites for PX-1. The lowest number of formed metabolites resulted from CYP2D6 (with 6) and CYP3A4 (with 5). The CYPs CYP1A2, CYP3A5, CYP2C19 and CYP2C9 formed 9–13 metabolites. Figure 4 shows the relative abundance of PX-1 metabolites with CYP incubation.

In comparison with pHLM incubation experiments, less metabolites were detected for *C. elegans* and CYPs. A total of 16 metabolites were observed by fungal incubation and a total of 26 metabolites were observed in experiments with CYP isoforms. Incubation with pHLM showed a total of 30 metabolites. Fungal incubation showed no exclusive metabolites. The comparison of formed metabolites by CYP, pHLM and *C. elegans* is shown in Table 3.

### Metabolic profile of PX-2

**Fig. 5:**
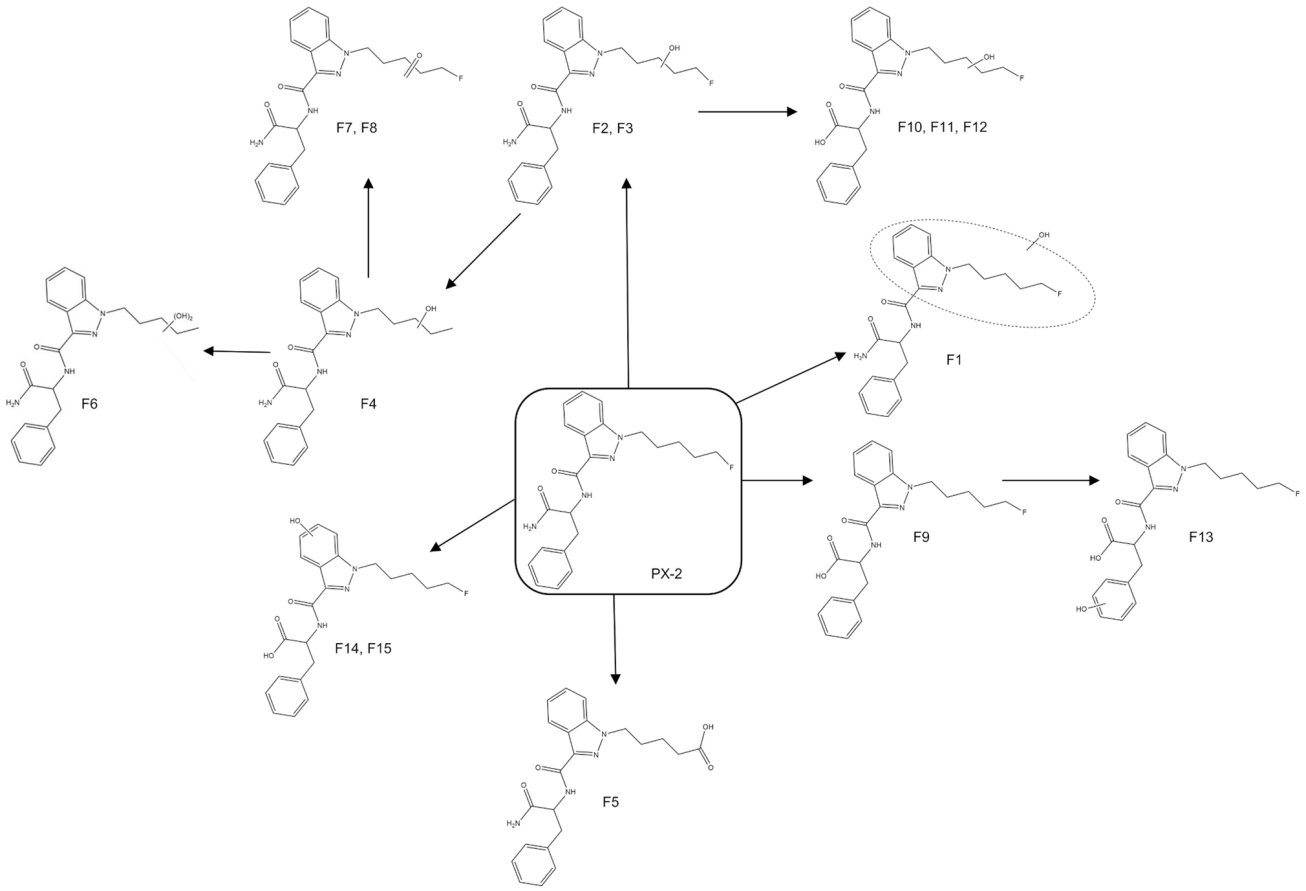
Proposed metabolic pathways of PX-2

After incubation with pHLM, CYPs and fungus, a total of 15 metabolites were identified for PX-2 (F1–F15), which are listed in Table 4. Furthermore, 3 metabolites (FU1–FU3) were also observed, the exact identity of which could not be determined with confidence. PX-2 peak area dropped to 11% after 1 h and 0.31% after 3 h pHLM incubation. Elution of metabolites occurred between 8.50 and 11.9  min. The parent compound eluted at 11.7 min. Metabolites were generated by monohydroxylation, oxidative defluorination, defluorination with mono- and dihydroxylation, carboxamide oxidation with subsequent oxidation and oxidative defluorination to carboxylic acid. Thirteen out of 15 detected metabolites were produced by pHLM. Fungal incubation of PX-2 showed a total of 11 metabolites; all these metabolites are identical with pHLM incubation. Incubation of PX-2 with CYP isoforms yielded 12 metabolites, with 12 metabolites being identical to those arising from pHLM incubation. An overview of metabolite structures and the predicted metabolic pathways of PX-2 is shown in Fig. 5.

**Table 4:** Comparison of the three in vitro models of PX-2 metabolism (pHLM, CYPs and fungus)

ID	Precursor ion [*m/z*]	Modification	Position	Rt [min]	pHLM	1A2	3A4	3A5	2B6	2C9	2C19	2D6	2E1	*C. elegans*
F1	413.19	Monohydroxylation	P or I	10.45	x	x	–	–	x	–	x	x	x	x
F2	413.19	Monohydroxylation	P	11.28	x	x	–	–	x	x	x	–	x	x
F3	413.19	Monohydroxylation	P	11.41	x	x	–	–	x	x	x	x	x	x
F4	395.20	Defluorination and monohydroxylation	P	11.27	x	–	–	–	–	–	–	–	x	–
F5	409.18	Carboxypentyl	P	10.32	x	–	–	x	–	x	x	–	–	x
F6	411.20	Defluorination and dihydroxylation	P;P	8.74	x	–	–	–	–	–	–	–	–	x
F7	411.20	Ketone	P	8.88	x	–	–	x	–	–	–	–	–	x
F8	411.20	Ketone	P	9.70	x	–	–	–	–	–	–	–	–	–
F9	398.19	Distal amide hydrolysis		11.88	x	x	x	x	x	x	x	x	x	x
F10	414.18	Distal amide hydrolysis and monohydroxylation	P	11.28	x	–	–	–	–	–	–	–	–	x
F11	414.18	Distal amide hydrolysis and monohydroxylation	P	11.40	x	x	–	–	–	–	–	–	–	x
F12	414.18	Distal amide hydrolysis and monohydroxylation	P	11.48	x	–	x	x	–	–	–	–	–	–
F13	414.18	Distal amide hydrolysis and monohydroxylation	B	11.52	x	–	–	–	–	–	x	–	–	x
F14	414.18	Distal amide hydrolysis and monohydroxylation	I	11.61	x	–	–	–	–	–	–	–	x	–
F15	414.18	Distal amide hydrolysis and monohydroxylation	I	11.66	x	x	–	–	–	–	–	–	–	x
PX-2	397.19	Parent compound		11.7	x	x	x	x	x	x	x	x	x	x

### Characteristic fragments of PX-2

**Fig. 6:**
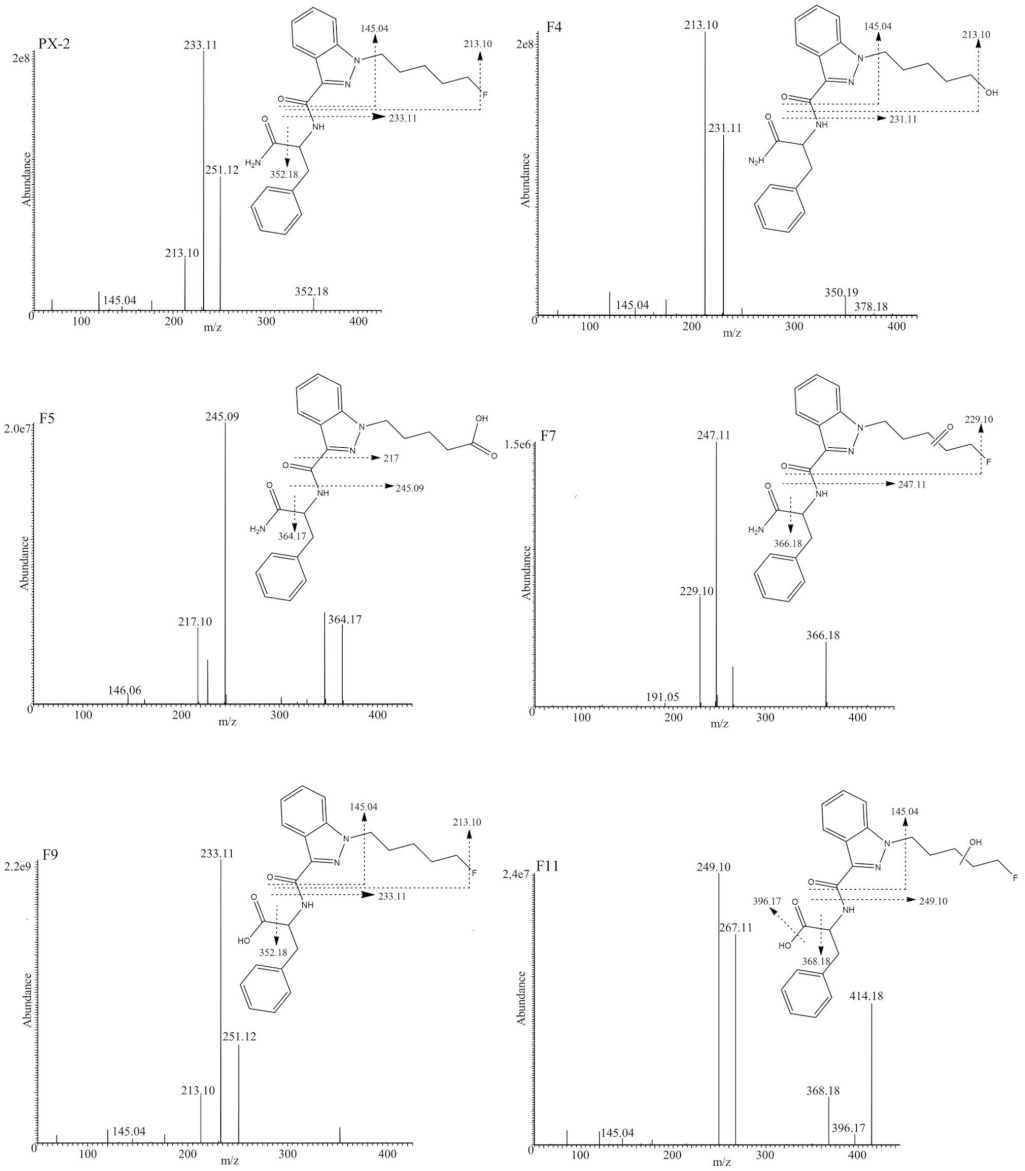
Product ion spectra of PX-2 and its five metabolites with proposed structures and fragmentation patterns

Five characteristic fragments were found for PX-2 in the product ion spectrum. Fragment *m/z* 233.11 is the unchanged fluoropentylindazole acylium ion, and removal of carboxamide group is demonstrated by *m/z* 352.18. For the observed product ion *m/z* 251.12, two structures can be considered: (a) a fluoropentylindazole acylium ion with addition of a water molecule as described by Fabregat-Safon et al. [13] and (b) a dealkylation of the fluoropentylindazole acylium ion with a shift of the phenyl group to the second indazole nitrogen as described by Cooman et al. [11]. Since a shift of the phenyl group to the second indazole nitrogen was considered to be less likely than the addition of water, configuration (a) was concluded for the first structure. Furthermore, the removal of the terminal carboxamide residue with penteneindazole acylium ion and unchanged indazole acylium ion yielded *m/z* 213.10 and 145.04 (see Fig. 6).

### Hydroxylated metabolites of PX-2

Oxidations similar to those observed for PX-1 occurred at different molecule sides. Three monohydroxylated metabolites (F1–F3) could be identified. Metabolite F1 is a result of monohydroxylation at the indazole core or the fluoropentyl side chain. The fragment *m/z* 249.10 (monohydroxylated fluoropentylindazole acylium ion) excludes hydroxylation at the benzyl moiety as it indicates monohydroxylation at the indole core or fluoropentyl side chain. Due to the absence of the characteristic fragments *m/z* 145.04 and 161.03 (monohydroxylated indazole acylium ion), it was not possible to determine where the monohydroxylation is located. Two pentyl side chain hydroxylated metabolites (F2 and F3) showed the key fragments *m/z* 249.10 and 145.04 (unaltered indazole acylium ion). Metabolite F4 resulted from hydroxylation after defluorination. The fragments *m/z* 231.11 (hydroxypentyl indazole acylium ion), 213.10 (pentyl  indazole acylium ion), and 145.04 indicate a monohydroxylation at the pentyl side chain. F4 was, therefore, considered to be a 5-hydroxypentyl metabolite.

Further hydroxylations occurred after defluorination, ketone formation, carboxylation and dihydroxylations. Oxidative defluorination to carboxylic acid was identified by the fragments *m/z* 245.09 and 217.10, which showed no hydroxylation at the indole core (F5). Two metabolites with ketone formation at the pentyl side chain could be detected (F7 and F8). This is suggested by the detected product ions *m/z* 229.10 for F7 and *m/z* 229.10 and 145.04 for F8. One defluorinated and dihydroxylated metabolite F6 were detected. Fragment’s *m/z* 247.11 (dihydroxylation of the pentylindazole acylium ion) and 366.18 (loss of the terminal carboxamide) indicated a defluorination and dihydroxylation for F6. The presence of fragments *m/z* 247.11 and 145.04, and the absence of fragment *m/z* 163.09, indicated a dihydroxylation at the pentyl side chain.

### Metabolites generated by amide hydrolysis of PX-2

**Fig. 7:**
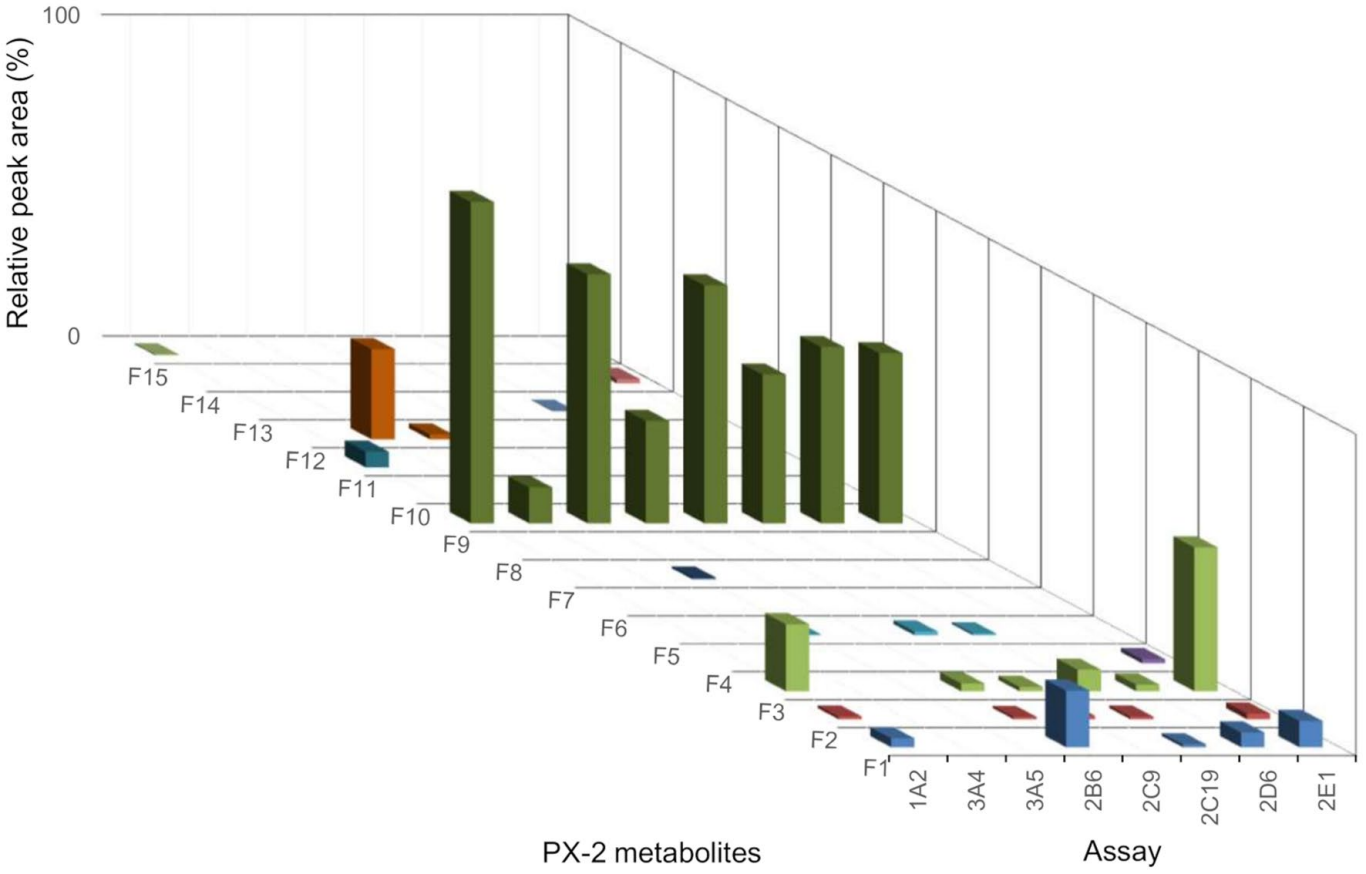
Results of the PX-2 CYP incubation. *X*-axis shows the different CYP isoforms, *z*-axis depicts the metabolite IDs and *y*-axis the relative peak areas in percent. Relative peak areas  were determined by dividing the raw peak areas of individual metabolites by the peak area of the most abundant metabolite identified

Likewise to observations made for PX-1 (Fig. 4), PX-2 terminal amide hydrolysis showed the highest abundance of all metabolites (Fig. 7). F9 shared common product ions with PX-2 and eluted 0.18 min after PX-1. Six metabolites could be detected with terminal amide hydrolyses and subsequent hydroxylation (F10–F15). The presence of the product ions *m/z* 249.10 and 145.04 indicated a monohydroxylation at pentyl side (F10, F11, and F12). Metabolite F13 is considered to be hydroxylated at the benzyl moiety. This is supported by observation of product ions *m/z* 233.11, 213.10, and 165.05 (C_9_H_9_O_3_^+^). Metabolites F14 and F15 showed the fragments ions *m/z* 249.10 and 161.03, which indicated hydroxylation at the indazole core.

### Unidentified metabolites (FU) of PX-2

In addition to the 15 identified metabolites, three further metabolites were detected, which, however, the molecular structure could not be assigned with confidence. For the metabolites FU1 ([MH^+^] *m/z* 413.19, Rt = 11.5 min) and FU2 ([MH^+^] *m/z* 413.19, Rt = 11.7 min), two further monohydroxylated metabolites are suggested by the observed parent ions. Metabolite FU3 ([MH^+^] *m/z* 395.20, Rt = 11.4 min) were considered to be a defluorinated and monohydroxylated metabolite that could only be detected by CYP isoforms 3A5, 2C19, and 2E1. FU1 and FU2 were detected in several CYP incubations and in the fungal incubation.

### Metabolic pattern of PX-2 after incubation with CYP and with *C. elegans*

PX-2 CYP metabolism differed from PX-1 in the number of metabolites. CYP1A2, CYP2E1 and CYP2C19 formed the most metabolites, with six metabolites. The CYPs, CYP3A5, CYP2B6 and CYP2C9 formed four metabolites. CYP3A4 and CYP2D6, with 2–3 metabolites, showed to play only a small role by the number of different metabolites in the metabolism of PX-2. Figure 7 shows the relative abundance of PX-2 metabolites with CYP incubation. The most abundant metabolite was F9, followed by F4, F1, and F12, which were produced by several CYPs. Fungal incubation with *C. elegans* showed less metabolites (11) than pHLM (17), and CYP (12) incubations. The comparison of formed metabolites by CYP, pHLM and C. elegans is shown in Table 4.

## Discussion

PX-1 and PX-2 were extensively metabolized by the applied in vitro assays, as expected from literature [11–13]. In contrast to previous publications on PX-1 and PX-2, in this study, a pHLM incubation was used for the investigation of metabolism as well as a CYP incubation, and a fungal incubation was applied [11–13]. These three in vitro assays were employed complementarily to obtain a better understanding of the in vitro PX-1 and PX-2 metabolic pathways. The published literature on the metabolism of PX-1 and PX-2 describes monohydroxylations, dihydroxylations, dealkylation, oxidative defluorination to carboxylic acid and amide hydrolysis with and without further oxidation. The most abundant metabolites found in the literature are the 5-hydroxypentyl metabolite, carboxylic acid metabolite and the dealkylated metabolite [11–13]. 5-Hydroxypentyl and carboxylic acid metabolites are well known for fluorinated synthetic cannabinoids such as MAM-2201, 5F-AB-PINACA and THJ-2201 [14, 17, 18, 38]. Our study findings are consistent with previously reported main metabolites. The most abundant PX-1 metabolites in our study were the 5-hydroxypentyl metabolite (M9), the amide hydrolyzed metabolite (M21), the distal amide hydrolyzed and defluorinated monohydroxylated metabolite (M29), and the *N*-dealkylated metabolite (M30), although there are slight differences with the published literature; for example, the amide hydrolyzed metabolite was not reported as a main metabolite. The differences in main metabolites between the previously published literature and our study could be explained by the differences in the experimental conditions of the performed in vitro assay [11–13]. It should also be noted that Fabregat-Safont et al. [13] were the only other group to detect the pentyl carboxylic acid with subsequent amide hydrolysis. The only publication with PX-2 metabolism was published by Cooman et al. [11]. We found the same metabolites as they did along with additional metabolites. The most abundant metabolite was, in both studies, the amide hydrolyzed metabolite (F9). The second most abundant metabolite in our study differs from Cooman et al. [11], as we detected this to be the defluorinated and monohydroxylated metabolite F4 instead of the defluorinated, pentyl-monohydroxylated and amide hydrolyzed metabolite. As PX-2 was highly metabolized to the amide hydrolyzed metabolite, this could have resulted in a lack of metabolite variety and lower signals for other metabolites. It is possible that the indazole core is mainly responsible for this observation. Unlike other SCs, such as AB-PINACA or 5F-AB-PINACA [14], epoxide or dihydrodiol metabolites could not be detected for both SCs in all in vitro assays. The oxidative deamination with another oxidation preferentially occurred over the dihydroxylation. With respect to the presented results and those described in the literature, the ketone formation seems to only play a minor role in PX-1 metabolism and could not be detected for PX-2 metabolism [11–13].

Unlike many other cannabinoids, PX-1 (indole core) is mainly, with regards to the number of different metabolites, metabolized by CYP2B6 and CYP2E1. CYP2B6 produced various metabolites high in abundancy. CYP2B6 played a minor part in the metabolism of other synthetic cannabinoids [18, 39]. PX-2 (indazole core) is mainly metabolized by CYP2E1, CYP2C19 and CYP1A2 (by the number of different metabolites produced). In contrast to its role in PX-1 and PX-2 metabolism, CYP2E1 has a small part in the metabolism of other cannabinoids [28, 36, 39–42]. When considering the abundance of metabolites produced by the CYP isoforms, PX-1 is mainly metabolized by CYP2B6 and CYP1A2, and PX-2 is mainly metabolized by CYP1A2 and CYP2E1.  SCs structurally similar to PX-1, such as UR-144 and XLR-11 as well as carbazole core cannabinoids EG-018 and EG-2201, were highly metabolized by CYP3A4 and CYP2D6 [28, 39, 40]. CYP3A4 played a small role in the metabolism of PX-1 and PX-2 with regards to the variety and abundance of metabolites (Figs. 4, 7). CYP2D6 generated a small variety of PX-1 and PX-2 metabolites, but produced M21 and F9 in comparably high abundancies. CYPs as CYP2C19, CYP2D6, CYP3A4, and CYP3A5 were observed to form dihydroxylated metabolites for UR-144, XLR-11, EG-018 and EG-2201 [39, 40]. In the case of PX-1, a signal corresponding to the dihydroxylated metabolite M16 was observed for most CYPs, but did not fit the peak identification criteria due to a signal-to-noise ratio < 3. SCs with an indazole core, such as AB-CHMINACA and THJ-018, were mainly metabolized by CYP3A4 and THJ-2201 by CYP2C19 [18, 39].

CYP isoforms CYP2B6, CYP2C9, CYP2C19, CYP2D6, CYP3A4 and CYP3A5 are mainly responsible for the formation of dihydroxylated metabolites for SCs with indazole cores [18, 28]. However, no dihydroxylated metabolite of PX-2 could be identified after CYP incubation. It is also possible that a concentration above 2% DMSO leads to a partial inhibition of CYP2C19 and CYP3A4 [43, 44]. In our study, we utilized 2.5% DMSO in the incubation; a possible reason for the reduced production of dihydroxylated metabolites can be, therefore, not completely ruled out. Monohydroxylation and oxidative deamination were formed by the most CYP isoforms for PX-1 and PX-2. Unsurprisingly, CYP1A2, CYP3A5, CYP2C19 and CYP2C9 also took part in the metabolism of PX-1 and PX-2, as previously observed for other cannabinoids [18, 36, 39–42].

Incubation with *C. elegans* showed a lower number of metabolites compared with pHLM and CYP incubation. The main metabolite of PX-1 (M9) and the main metabolite of PX-2 (F9) could also be found in fungal incubation. The metabolism of *C. elegans* seems not to catalyze *N*-dealkylation. For 10 SCs incubated with *C. elegans,* only three showed *N*-dealkylated metabolites (UR-144, EG-018 and EG-2201) [23, 30, 34, 40]. In the case of dihydroxylated metabolites, fungal incubation exhibited more dihydroxylated metabolites than CYP incubation. As expected, the major monohydroxylated metabolites were found with fungal incubation. Likewise to pHLM and some CYPs, ketone formation was also detected with *C. elegans.* The literature described SCs metabolites without ketone formation for 5F-PB and XLR 11 for incubation with *C. elegans* [34]. *C. elegans* showed a variety of different metabolites and was in good agreement with the metabolic profile observed with pHLM and cytochrome P 450 isoforms. In particular, the most abundant metabolites detected with the other assays were mainly detectable after incubation with *C. elegans.* Therefore*,* metabolism with *C. elegans* was determined to be an especially useful (alternative) tool for in vitro study of SCs, as has been described in literature for other SCs [23, 30, 34, 35, 40]. Its advantages are particularly evident in its reduced costs, as one culture could be easily/continually cultivated.

## Conclusions

This study presents the in vitro phase I metabolism of PX-1 and PX-2. A total of 30 metabolites for PX-1 and 15 metabolites for PX-2 were identified by pHLM, CYP and fungal incubation. The metabolites of PX-1 and PX-2 identified by pHLM incubation were generally in accordance with CYP and fungal incubation, and with the previously published metabolites of PX-1 and PX-2. The 5-hydroxypentyl metabolite (M9) and the amide hydrolyzed metabolite (M21) were the most abundant metabolites of PX-1. Metabolite F9 (PX-2 analog of M21) and defluorinated and monohydroxylated metabolite F4 were the most abundant metabolites for PX-2. These  metabolites are promising detection markers, as suggested by previously published PX-1 and PX-2 studies [11–13]. Furthermore, the metabolites M21 and F9 could be also used to distinguish PX-1 and PX-2 from their non-fluorinated analogs APP-PICA (PX-1 analog) and APP-PINACA (PX-2 analog). It is also noticeable that PX-1 and PX-2 were mainly metabolized by CYP2E1 and CYP2B6, while PX-2 was metabolized by CYP2E1 and CYP1A2 instead of CYP3A4, which is the main CYP for many other cannabinoids. Regarding the abundancy, PX-1 is mainly metabolized by CYP2B6 and CYP1A2 and PX-2 by CYP1A2 and CYP2E1. Fungal incubation showed, as expected, a good correlation with the pHLM and CYP incubation. The involvement of various CYPs in the metabolism of PX-1 and PX-2 (especially in the formation of the highly abundant metabolites M21 and F9) indicates that metabolic drug-drug interaction via competition for the metabolizing CYP, is very unlikely for PX-1 and PX-2.

This study shows that all three in vitro assays could be used for the prediction of metabolic pathways of SCs. To establish the completeness of the PX-1 and PX-2 metabolic pathways, it is necessary to use different assays. To reveal the in vivo relevance of the metabolites, in vitro-observed metabolite spectra must be compared with the metabolic pattern found in authentic urine samples. Still, the combination of in vitro assays provides a powerful tool for metabolite integration into screening methods, allowing the establishment of detection methods prior to testing real samples. The differences observed in the metabolism of PX-2, with regards to high biotransformation to the amide hydrolyzed metabolite instead of the formation of a large variety of different metabolites, should be considered in further metabolism studies of structurally-related SCs.
